# Phylogeography and Re-Evaluation of Evolutionary Rate of Powassan Virus Using Complete Genome Data

**DOI:** 10.3390/biology10121282

**Published:** 2021-12-06

**Authors:** Artem N. Bondaryuk, Tatiana E. Peretolchina, Elena V. Romanova, Anzhelika V. Yudinceva, Evgeny I. Andaev, Yurij S. Bukin

**Affiliations:** 1 Laboratory of Natural Focal Viral Infections, Irkutsk Antiplague Research Institute of Siberia and the Far East, Irkutsk 664047, Russia; e.andaev@gmail.com; 2 Department of Genosystematics, Limnological Institute, Siberian Branch of the Russian Academy of Sciences, Irkutsk 664033, Russia; tatiana.peretolchina@gmail.com (T.E.P.); elena_romanova@lin.irk.ru (E.V.R.); udinceva.a@yandex.ru (A.V.Y.); bukinyura@mail.ru (Y.S.B.)

**Keywords:** Powassan virus, Bayesian inference, phylogenetics, phylogeography, evolutionary rate

## Abstract

**Simple Summary:**

The evolution of human pathogenic viruses is one of the pressing problems of modern biology and directly relevant to public health. Many important aspects of virus evolution (e.g., evolutionary rate, population size, and migration history) are ‘hidden’ from the naked eye of a researcher. Modern bioinformatics methods make it possible to evaluate and visualize such evolutionary particularities of viruses. In this paper, we reconstructed the migration history and estimated the evolutionary rate of one of the most dangerous neuroinvasive and neurotropic tick-borne flaviviruses—Powassan virus (POWV)—distributed in North America and the Far East of Russia. Using the dates obtained, we hypothesized that the divergence of the most recent common ancestor of POWV into two independent genetic lineages most likely occurred because of the melting of glaciers that began at 11.72 Kya in the Holocene due to the climate warming-caused flooding of the isthmus between Eurasia and North America.

**Abstract:**

In this paper, we revealed the genetic structure and migration history of the Powassan virus (POWV) reconstructed based on 25 complete genomes available in NCBI and ViPR databases (accessed in June 2021). The usage of this data set allowed us to perform a more precise assessment of the evolutionary rate of this virus. In addition, we proposed a simple Bayesian technique for the evaluation and visualization of ‘temporal signal dynamics’ along the phylogenetic tree. We showed that the evolutionary rate value of POWV is 3.3 × 10^−5^ nucleotide substitution per site per year (95% HPD, 2.0 × 10^−5^–4.7 × 10^−5^), which is lower than values reported in the previous studies. Divergence of the most recent common ancestor (MRCA) of POWV into two independent genetic lineages most likely occurred in the period between 2600 and 6030 years ago. We assume that the divergence of the virus lineages happened due to the melting of glaciers about 12,000 years ago, which led to the disappearance of the Bering Land Bridge between Eurasia and North America (the modern Alaskan territory) and spatial division of the viral areal into two parts. Genomic data provide evidence of the virus migrations between two continents. The mean migration rate detected from the Far East of Russia to North America was one event per 1750 years. The migration to the opposite direction occurred approximately once per 475 years.

## 1. Introduction

Powassan virus (POWV) was isolated for the first time in September 1958 in Canada (Powassan, Ontario) from a child with fatal encephalitis [1].

POWV is a tick-borne flavivirus transmitted by *Ixodes* spp. (e.g., *I. marxi*, *I. scapularis*, *I. cookie*, *I. persulcatus*, etc.) [2], which is spread across North America (NA) and the Russian Far East (RFE), and causes a fatal neuroinvasive disease in humans [3]. Approximately 10% of POWV encephalitis cases are fatal, and 50% cause severe and long-lasting neurological sequelae [4]. The mortality of the most other neuroinvasive tick-borne flaviviruses is lower. For instance, the European subtype of tick-borne encephalitis virus causes about 1–2% of fatal cases, and the louping-ill virus has only one fatal case registered [5]. Among the variants of POWV in NA, the subtype “Deer tick virus” (DTV) is distinguished based on genetic characteristics, the area distribution, and the spectrum of vectors [3].

The genome length of the POWV is approximately 11,000 nt. The largest part of the sequence (10,248 nt.) encodes a single polyprotein (3416 a.a.), which is cleaved co- and post-translationally into three structural proteins (C, prM, and E) and seven non-structural proteins (NS1, NS2A, NS2B, NS3, NS4A, NS4B, and NS5) [6].

The evolutionary rate of POWV and time of the most recent common ancestor (tMRCA) were assessed in previous studies using different approaches based on Bayesian inference [7,8]. However, in the phylogenetic studies performed in the early 2010s, researchers often did not evaluate the temporal signal (a sufficient number of genetic changes between sampling times) [9] that particularly reduces the validity of the analysis, especially when incomplete genomic sequences and a narrow sampling window relative to total tree length are used [10].

Another pitfall of that king of analysis is that Bayesian phylogenetic software (e.g., BEAST) can generate nodes with high posterior support even when the data does not have a significant temporal signal. Therefore, the results of the previous studies of the POWV evolutionary rate and tMRCA estimation need more accurate assessment.

Other interesting aspects of POWV molecular epidemiology are the phylogeographic relationships between virus isolates. In previous genetic studies, researchers hypothesized that POWV could be introduced into the RFE from NA [11,12]. However, such studies used relatively small data sets maintained either partial gene sequences or a small number of complete genome sequences. A significant sequence data accumulation and the development of algorithms of phylogenetic analysis in recent years allow researchers to obtain more robust results. In particular, a new approach of structured coalescent approximation implemented in the MASCOT package [13] of the BEAST 2 [14] allows the assessment of phylogeographic relationships between virus populations with the dating of migration events by the times of the strains’ isolation. This method can be helpful for estimation in detail of the POWV distribution in the areas of its natural foci. As of June 10, 2020, 26 complete genomes of POWV with known isolation dates (1958–2019) are available in NCBI and ViPR. This number of complete genomes should be sufficient for dating the main events of the viral evolution in the phylogeographic analysis.

In this study, we aim to estimate temporal signal in the set of the POWV complete genomes, re-evaluate the evolutionary rate of the virus and reveal the phylogeographic relationships between POWV isolates. We also suggest a new simple Bayesian technique for the evaluation and visualization of ‘temporal signal dynamics’ along the phylogenetic tree. This technique would be an especially useful tool for phylogenetic analysis in cases when a sampling window is small relative to tree length that leads to underestimating the temporal signal of a whole tree even when tMRCA of young clades have enough signal to be calculated reliably.

## 2. Materials and Methods

### 2.1. Genomic Data Sampling

Nucleotide sequences of a POWV polyprotein gene (10,248 nt) were downloaded from NCBI and ViPR [15] databases (accessed in June 2021. In total, 26 sequences were used in the analysis. The dates of their isolation were taken from the literature and covered the period from 1958 to 2019 (Table 1).

Eight sequences were isolated in the RFE (Russian Far East) and the others were from NA (North America) (one from Canada and 17 from the USA). The RFE and NA groups were used as ‘states’ in the phylogeographic analysis.

### 2.2. Codon Saturation Measuring and Evolutionary Model Selection

Index of substitution saturation (I_ss_) in a set of aligned nucleotide sequences was calculated in DAMBE [16] and compared with critical I_ss_ value (referred to as I_ss.c_ [17]).

The best-fit substitution model was chosen according to the BIC score estimated by ModelFinder [18] implemented in IQTREE v1.6.12 [19].

The selection between strict and relaxed molecular clock was made based on the value of the coefficient of substitution rate variation (CV) obtained in a preliminary BEAST v2.6.3 run [14]. A total of 200 million MCMC generations were run, where every 5000th generation was sampled, and the first 10% of generations were discarded as burn-in. The effective sample size (ESS) for all parameters exceeded 200. A relaxed clock model with an uncorrelated lognormal distribution (UCLD) of substitution rates and constant population size prior were used. If 95% highest posterior density (HPD) of CV maintains a zero value, the strict molecular clock is used for the data set [20].

The consensus tree and the branch rate heterogeneity were visualized in FigTree.

### 2.3. Assessment of Temporal Structure of Heterochronous Sequences

The temporal signal in the data set was evaluated using the Bayesian model test—BETS (Bayesian Evaluation of Temporal Signal) where times of virus isolation were used as one of the model priors [10]. 

To perform BETS the marginal likelihood values for heterochronous and isochronous models (*M_het_*—a model with dated tree tips in BEAUti and *M_iso_*—a model without tips dating) should be calculated by any appropriate method. We evaluated marginal likelihood values using Path Sampling (PS) in BEAST 2 and ranked them according to their log Bayes factors (log BFs). Log BFs were interpreted according to the guidelines of Duchene, et al., (2020) [10], where a difference in log BF between models more than 5 indicates ‘very strong’ support.

We performed PS analysis both for the model with the correct sampling times (heterochronous data) and for the model without sampling times (isochronous tree). For both models, we used a strict clock and constant size model as a coalescent tree prior. For each model we set three PS runs with 200 steps per run. A total of 10 million MCMC iterations were set as pre-burning step, and 500,000 iterations were set for each step. The sufficient lengths of MCMC chains inside PS step and number of steps were defined by comparing the log marginal likelihood values obtained in different runs. If the difference among log marginal likelihood values in the runs was not significant, the parameters acknowledged to be sufficient for convergence. For more detail about prior settings for BETS, see Table S1.

Additionally, we performed a permutation test with 20 different random permuted between sequences isolation dates. For each of the 20 permutations, a BEAST analysis was performed with the selected parameters of the evolution model and molecular clock. In these analyses, tree root height and evolutionary rate values were determined. The results obtained were then compared with the analysis with correct isolation dates. If the root height and evolutionary rate values do not differ from the analysis with correct isolation dates, it indicates the absence of the temporal signal in the genomic dataset studied.

We additionally performed a permutation test to detect the temporal signal in the data set. We carried out 20 different random permutations between sequences isolation dates. For each permutation, BEAST analysis was performed with the selected parameters of the evolution model and molecular clock. If the root height and evolutionary rate values obtained in the permutation test do not differ from the results of the analysis using correct viral isolation dates, it means the absence of the temporal signal in the genomic dataset studied.

### 2.4. Temporal Signal Dynamics

Considering a narrow sampling window and relatively ancient root age, theoretically, the signal can be absent near the root and appear closer to younger clades [10].

**Figure 1:**
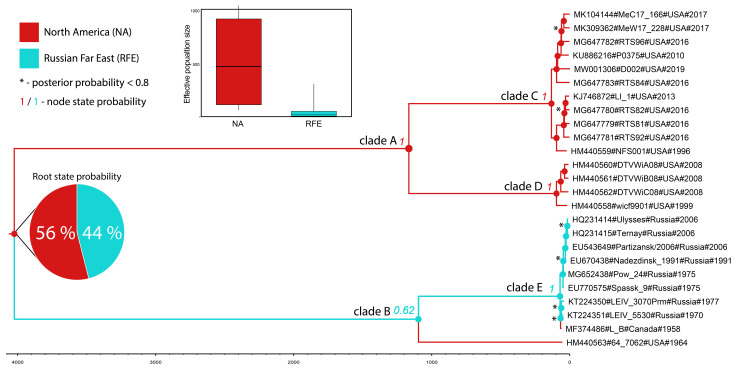
The time scaled phylogenetic tree of POWV in NA and FER: The color of tree branches and nodes represent a state/territory (see legend in the top left corner). The change in colors is a virus migration event between the states/territories. Numbers to the left from main nodes are node state/territory posterior probability. Black asterisks show clades with posterior probability less than 0.8 (please, do not confuse with node state/territory posterior probability). In the other cases, posterior probability is more than 0.8. An insert is the relative genetic diversity (effective population size (N_e_) × generation time (τ—time between two successful infections in the chain of transmission).

To illustrate possible temporal signal dynamics along the POWV phylogenetic tree, we sampled tMRCA values from the prior and posterior distributions for the root and the first five clades of the tree were designated as A, B, C, D, and E clades (Figure 1).

The step-by-step tutorial prepared by us with a complete description of the temporal signal dynamics analysis scheme is available from: https://doi.org/10.6084/m9.figshare.16782484 (accessed on 6 December 2021).

### 2.5. Phylogeographic Analysis

The phylogeographic structure of POWV was studied using a structure coalescent approach and MASCOT package v1.2.2 [13] implemented in BEAST 2. The MASCOT was chosen as a tool capable of performing a robust analysis under biased sampling [21].

The number of MCMC generations for the MASCOT analysis was 100 million, where each 5000th entry was sampled (the total number of samples was 20,000).

## 3. Results

### 3.1. Assessment of Codon Substitution Saturation and the Best-Fit Substitution Model

Analysis of POWV polyprotein gene set in DAMBE [16] did not reveal substitution saturation both in the first and second codon positions (I_ss_ = 0,10 and I_ss.c_ = 0.82 at *p*-value = 0.00), and in the third codon position (I_ss_ = 0.25 and I_ss.c_ = 0.80 at *p*-value = 0.00), (Table S2), indicating this set appropriate for phylogenetic study.

The GTR + I was chosen as the best substitution model according to the BIC score value (see Table S3) and used for further BEAST analysis.

### 3.2. Molecular Clock Choosing

The preliminary run (GTR + I + UCLD + Constant size model) using 26 POWV sequences set revealed an unusually high value of the coefficient of variation (95% HPD, 0.25–1.47), considering that slowly evolving tick-borne flaviviruses do not have a high-rate variation among tree branches, as it was shown, for instance, for tick-borne encephalitis and louping-ill virus by Uzcátegui, et al., (2012) [22].

A branch of ‘ctb30’ strain on the consensus tree has an extraordinarily high evolutionary rate (1.6 × 10^−4^ substitutions per site per year) in comparison with the mean rate across the whole tree (Figure S1).

Such a high evolutionary rate value may be explained either with the longer passage history of the ‘ctb30’ strain (three times in suckling mice and two times in tissue culture) or by the errors in sequencing. Thus ‘ctb30′ strain was excluded from the analysis. 

A new run of 25 sequence set led to the narrowing of 95% HPD (0–0.39), where the majority of values distributed around zero and a zero value was the lowest boundary of 95% HPD (Figure S2). Thus, the strict molecular clock model was chosen for further analysis of temporal signal assessment in this data set.

### 3.3. Comparing Prior and Posterior Distributions. Temporal Structure and Temporal Signal Dynamics

The BETS approach yielded very strong support for heterochronous over isochronous models (the lowest log BFs for SC_het_ in the case of SC_het_ over SC_iso_ in all three runs were 30, see Table 2). This result indicates that the data unequivocally contain temporal signal.

**Figure 2:**
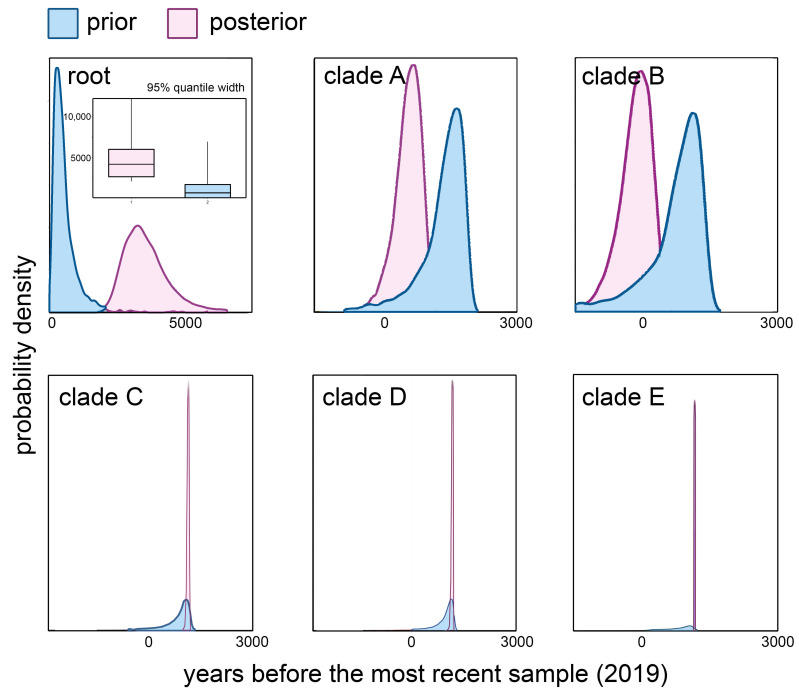
The prior and posterior distributions of tMRCAs of root age and five main clades (clades (**A**–**E**)). An insert within the root plot is boxplots of 95% quantile width of the tree root height.

The temporal signal near the tree root was indicated by the non-overlapping 95% quantile distributions of the root age values obtained for prior and posterior data (Figure 2, root).

In clades A and B (Figure 2, clades A, B) the 95% quantile width of prior and posterior distribution were overlapped, but the posterior distribution array was shifted from prior, which indicates the minor impact of temporal signal on tMRCAs in these clades.

Comparison of prior and posterior for tMRCA values of clades C, D, and E revealed that the temporal signal becomes stronger closer to the tips: posterior densities were much narrower than prior (Figure 2, clades C, D, and E).

### 3.4. Permutation Test

**Figure 3:**
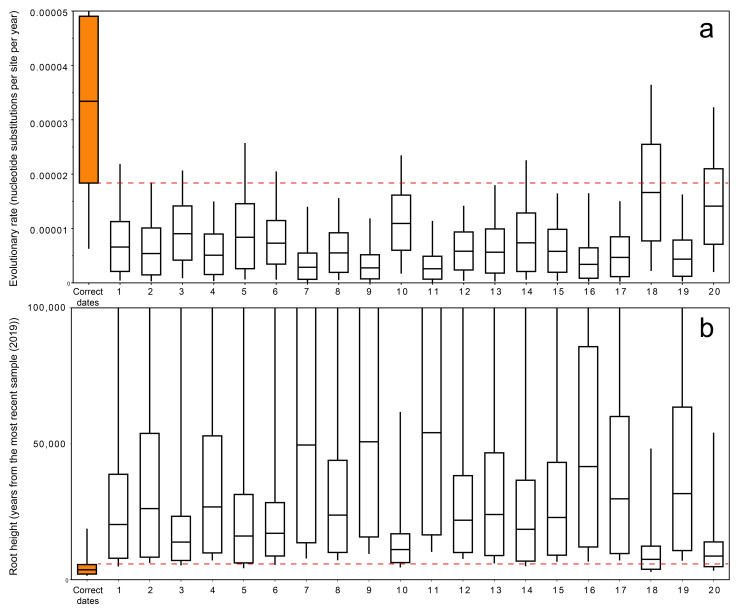
Date permutation test results: The boxplots present 95% HPD of (**a**) evolutionary rate and (**b**) tree root height parameters. Analysis with correct isolation dates is shown as the orange bar. Horizontal dashed lines demonstrate overlapping areas of the analysis using correct dates and permuted replicates.

Permutation test showed that in 18 out of 20 runs, 95% HPD intervals of estimated evolutionary rates and tree heights did not overlap with intervals of values obtained considering the real isolation dates of viruses (Figure 3a). A similar result was shown for the root height values, where 95% HPD intervals of only two permuted runs were overlapped with values estimated from the real isolation dates (Figure 3b). Generally, it is consistent with the BETS results.

### 3.5. Evolutionary Rate Estimates

The mean evolutionary rate of a POWV polyprotein gene estimated by SC was 3.3 × 10^−5^ nucleotide substitutions per site per year (95% HPD, 2.0 × 10^−5^–4.7 × 10^−5^).

### 3.6. Divergence Dating

We specify here the dates of the root and nodes divergence as years dated back from the year of the most recent sample isolation (2019).

The tree root diverged in 4170 (95% HPD, 2600–6030). The mean divergence times for the clades A and B were 1160 (95% HPD, 760–1750), and 1100 years (95% HPD, 721–1619), respectively (Figure 1). 

According to the analysis, the time of the root divergence is 4170 years (95% HPD, 2600–6030). The mean divergence times for the clades A and B are 1160 years (95% HPD, 760–1750), and 1100 years (95% HPD, 721–1619), respectively (Figure 1).

TMRCAs of the clades C, D, and E, are 135 years (95% HPD, 87–200), 97 years (95% HPD, 62–143), and 71 years (95% HPD, 62–87), respectively.

### 3.7. Phylogeography

We obtained an unequivocal assessment of the root state using MASCOT. The structured coalescent inference revealed similar probability values for both territories of the POWV spread (56% and 44% for NA and RFE, respectively; Figure 1). The estimation of the clade B node state was more reliable (62% and 38% for RFE and NA, respectively; Figure 1); however, it remained ambiguous. Other tree nodes have state probability of one.

The relative genetic diversity (Ne × τ) estimated for NA POWV was about 622, which is significantly higher than the appropriate value for RFE POWV (≈25). The longer internode patristic distances between the NA POWV isolates of clade A in comparison with that of clade B supports the data about the increased genetic diversity of the first one.

The migration of POWV occurs in both directions. The mean rate for RFE to NA migration is one event per 1750 years, and migrations in the opposite direction occur once per 475 years, respectively.

## 4. Discussion

The evolutionary rate of POWV (95% HPD, 2.0 × 10^−5^–4.7 × 10^−5^ substitutions per site per year) estimated in this work was much lower than values reported in the previous studies, based on the sequences of single genes or their fragments. Pesko, et al., (2010) [7] estimated the rates of accumulation of nucleotide substitutions in the genome region encoding proteins E and NS-5 to be 2.2 × 10^−4^ and 3.9 × 10^−5^ substitutions per site per year, respectively. Subbotina and Loktev (2012) [8] showed that rate of nucleotide substitutions per site per year for the E protein was 1.4 × 10^−4^ and for NS-5 it was 5.4 × 10^−5^. Thus, the previous values of the evolutionary rates for region encoding protein E were almost 10 times higher than our estimates for the coding part of the POWV genome. For the fragment encoding NS-5 protein, the rate estimates were close or slightly overlapped the upper limit of the confidence interval we obtained. Such an increased evolutionary rate in previous studies could have been obtained due to the absence of a preliminary evaluation of temporal signal, and the effect of priors, specified in BEAUti (the program used to design the analysis and generate the BEAST 2 project file).

The evolutionary rate of POWV estimated in this study is compatible with that of TBEV and LIV (1.0 × 10^−5^–2.2 × 10^−5^ for TBEV and 5.7 × 10^−6^–3.9 × 10^−5^ for LIV [22,23,24]). Tick-borne flaviviruses, such as POWV, TBEV, and LIV, are known as slowly evolving RNA-viruses. Many other RNA-viruses have one or two orders of magnitude higher substitution rates. For instance, the substitution rate in measles virus is 3.6 × 10^−4^–9.0 × 10^−4^ [25]; in Hepacivirus C virus is 9.0 × 10^−4^ [26]; Zika virus is 6.2 × 10^−4^–8.2 × 10^−4^ [27]; HIV-1 is 1 × 10^−3^–1.7 × 10^−3^ [28]; and SARS-CoV-2 is 1.3 × 10^−3^–2.0 × 10^−3^ [29].

The possible reasons for the lower evolutionary rate of tick-borne flaviviruses (POWV, LIV, and TBEV) in comparison with mosquito-borne flaviviruses (e.g., dengue virus, 8.9 × 10^−4^ substitutions per site per year [30]), and other RNA-viruses without arthropod vectors (e.g., influenza virus and HIV-1) may be the features of the tick vector biology. Tick–host interaction is a two-host system which imposed constraining effect on the virus evolution. Moreover, ticks have an extended life-cycle (3–5 years), as compared with mosquitoes, and accumulate the virus in the relatively low titers which, thereby, reduces the viral evolutionary rate [31]. 

Clade E, comprising all isolates from FER and one isolate from Canada, was recognized as the youngest virus group (95% HPD, 62–87). Moreover, tMRCA of the clade E was assessed most accurately as it has the narrowest 95% HPD. The chronology of the occurrence between clades D vs. C and D vs. E remains vague, because of the overlap of 95% HPD intervals (Figure S3).

The previous study of Leonova, et al., (2009) [12] proposed several hypotheses of POWV transmission from Canada to the Russian Far East. The hypothesis of the human-caused transmission of TBEV in the 20th century (the parental node of clade E—95% HPD, 61–82 years ago) by the transition of military cargo, troops, animals, etc., was admitted as more likely, than one of natural transmission by the wild birds. However, according to our estimations, the POWV split into the NA and FER genetic lineages, which happened in the period between 2600 and 6030 years ago (the mean value is 4170 years ago, the root state reconstruction), has the natural reason, such as global climatic changes. The ambiguity in the evaluation of the root state could be explained by the POWV MRCA origination at the border area in Beringia.

The geological studies showed that the Bering Land Bridge connected Eurasia (its FER part, in particular) and North America in the Pleistocene during the last glaciation from 11.72 to 30 thousand years ago [32,33]. So, Beringia could be a migration route for people, animals, and plants from Eurasia to North America and vice versa. The evidence of those migrations was obtained in population genetic studies of different animal species [34,35,36]. MRCA of the modern POWV could exist on the territory of RFE, NA, and Beringia as a single viral population. The main vectors of POWV are *Ixodes cookie* and *I. scapularis* ticks, which parasitize on deer and small rodents [37], which in turn were abundant in RFE, NA, and Beringia during the last Ice Age. The melting of glaciers began at 11.72 Kya in the Holocene due to the climate warming caused flooding of the isthmus between Eurasia and North America [38], and the gene flow between the two POWV groups decreased radically. From that time, the formation of two genetic lines of the virus started and was completed about 4000 years ago. However, the genetic contacts between RFE and NA groups of POWV did not entirely quit. Rare virus transmission events have occurred from 4000 years ago to the present. According to data of Ogden, et al., (2008) [39], *I. scapularis* can parasitize on migratory birds. Thus, the migratory birds carrying infected ticks may provide gene flows between POWV from RFE and NA.

The results of the analysis of migratory events between the RFE and NA POWV groups reported in our study are not entirely reliable, and a more detailed study requires a significant increase in the sample size of the genomes both in NA and RFE. However, our work results lead to an unambiguous conclusion about the presence of such recent migration events between the two POWV groups.

Some studies of viruses as well as organisms of other groups showed that in many cases there is the possibility of underestimating the age of the root of the phylogenetic tree using molecular clocks due to the effect of slowing down the rate of the molecular clock in the tree root area [40,41] and/or fixing adaptive mutations during the speciation and the development of a new ecological niche [42]. Wertheim, et al., (2013) [42], using the origin of corona viruses as an example, showed that when viruses change their hosts, a distortion of the molecular clock occurs with an underestimation of the age of ancient evolutionary events. In our case, when POWV separated from the closest ancestors, it assimilated a new ecological niche with a change in the host spectrum. Therefore, the divergence time of the POWV lineages 4000 years ago may be underestimated. So, the evolutionary events caused the formation of two POWV clades on the territory of RFE, NA, and Beringia may have occurred at 11.72 Kya.

The phylogeographic structure of POWV isolates showed in our study corroborates the conclusions of Heinze, et al., (2012) [11], where the glaciation is hypothesized as the main factor that contributed to the divergence POWV. The authors [11], also considered the POWV movement from Old World to New World, and strongly suggested southern Asia be a source of POWV. Our results make it possible to assume that the POWV ancestor most likely entered in its ancient area (FER, Beringia, and NA) from the Old World—the region of the origin of flaviviruses [11]. Therein, this ancestor, adapting both to the local conditions and spectrum of hosts (ticks and warm-blooded animals), evolved into POWV. Thus, we confirm the findings of Heinze, et al., (2012) [11].

The larger values of relative genetic diversity (≈615) of the NA POWV population in comparison with RFE POWV (≈25; Figure 1) imply better conditions for virus replication and spread in the NA territory. This may be explained by the absence of competition from the other tick-borne flaviviruses (e.g., TBEV distributed in Asia and Europe). POWV occupied the free local vectors (*I. cookie* and *I. scapularis*) and spread across the northern part of NA. In the RFE region, POWV theoretically can compete with TBEV via a cross-immune response in warm-blooded animals. Experiments have shown [43,44,45] that a wide range of flaviviruses, including POWV and TBEV, have partial cross-neutralization of viral particles by antibodies. Thus, if a warm-blooded animal has ever encountered TBEV and received an immune response, it can break the cycle of transmission of the POWV in the tick–warm-blooded animal–tick transmission chain. Therefore, POWV apparently loses in the competition to the more abundant TBEV in the given territory that leads to decreasing of POWV genetic diversity. On the other hand, the possible effect of a small sample size for POWV in the RFE territory, which can randomly distort the index of genetic diversity, should not be excluded. A more detailed answer to this question requires the collection of additional experimental materials.

The MASCOT revealed asymmetric migration rates between states, with more intense migration from NA to RFE direction. It is important to note that we assessed the divergence of the ancestral POWV population since the Beringia flood, and did not cover more ancient periods of viral population history. Thus, there is no contradiction between the conclusions in our work and that obtained by Heinze, et al., (2012) [11] about southern Asia being the source of tick-borne flaviviruses.

## 5. Conclusions

The mean evolutionary rate inferred for POWV based on Bayesian analysis of complete genome data was 3.3 × 10^−5^ nucleotide substitutions per site per year (95% HPD, 2.0–4.7), which is comparable with the estimates of substitution rate of other tick-borne flaviviruses such as TBEV and LIV, and significantly lower than the rate estimates yielded previously. The analysis of temporal dynamics showed the presence of a temporal signal in all branches of the POWV phylogenetic tree with the strongest data impact on the tMRCA evaluation of three clades (C, D, and E) (95% HPD intervals 87–200, 62–143, 62–87, respectively). 

The MRCA of modern POWV split into two independent genetic lineages between 2600 and 6030 years ago as a result of the Beringia flood about 11.72 thousand years ago.

The absence of competition from other tick-borne flaviviruses for the free local vectors (*I. cookie* and *I. scapularis*) in NA had a positive impact on the higher genetic level of the genetic diversity of NA POWV (≈615) comparing with the RFE virus population (≈25). However, the difference in relative genetic diversity between NA and RFE populations of POWV may be caused by the lack of sequence data on the territory of Russia. Thus, in case of further RFE POWV isolation and genome sequencing, the accuracy of phylogeographic analysis can be increased.

## Figures and Tables

**Table 1:** Sequences’ metadata.

GB_ID	Strain	Commentary	Country	Year	Host
MF374486	L_B		Canada	1958	Human
KT224351	LEIV_5530		Russia	1970	*Ixodidae* spp.
EU770575	Spassk_9		Russia	1975	*Dermacentor silvarum*
MG652438	Pow_24		Russia	1975	*I. persulcatus*
KT224350	LEIV_3070Prm		Russia	1977	*Ixodidae* spp.
EU670438	Nadezdinsk_1991		Russia	1991	Human
HQ231415	Ternay		Russia	2006	Human
EU543649	Partizansk/2006		Russia	2006	Human
HQ231414	Ulysses		Russia	2006	Human
HM440563	64_7062		USA	1964	Tick
AF311056	ctb30	DTV subtype of POWV	USA	1995	-N/A-
HM440559	NFS001		USA	1996	*I. scapularis*
HM440558	wicf9901	DTV subtype of POWV	USA	1999	*I. scapularis*
HM440561	DTVWiB08	DTV subtype of POWV	USA	2008	*I. scapularis*
HM440560	DTVWiA08	DTV subtype of POWV	USA	2008	*I. scapularis*
HM440562	DTVWiC08	DTV subtype of POWV	USA	2008	*I. scapularis*
KU886216	P0375		USA	2010	*I. scapularis*
KJ746872	LI_1		USA	2013	*I. scapularis*
MG647783	RTS84		USA	2016	*I. scapularis*
MG647781	RTS92		USA	2016	*I. scapularis*
MG647782	RTS96		USA	2016	*I. scapularis*
MG647779	RTS81		USA	2016	*I. scapularis*
MG647780	RTS82		USA	2016	*I. scapularis*
MK309362	MeW17_228		USA	2017	*I. scapularis*
MK104144	MeC17_166		USA	2017	*I. scapularis*
MW001306	D002		USA	2019	Human

**Table 2:** Log marginal likelihood values for heterochronous and isochronous models estimated by path sampling (PS) in BEAST 2.

Model	Log marginal Likelihood		
	PS Run 1	PS Run 2	PS Run 3
SC_het_ ^1^	−26,773.5	−26,771.5	−26,771.1
SC_iso_	−26,808.8	−26,808.5	−26,807.6

^1^ The most appropriate model with the highest log marginal likelihood values.

## Data Availability

Data supporting reported results (nucleotide data sets, BEAST projects) are available from: https://doi.org/10.6084/m9.figshare.16782484 (accessed on 6 December 2021).

## Supplementary Materials

The following are available online at https://www.mdpi.com/article/10.3390/biology10121282/s1, Figure S1: Evolutionary rate heterogeneity, Figure S2: Comparison the coefficient of rate variation values, Figure S3: Divergence time of tMRCAs of the three main clades, Table S1: BETS priors for the different BEAST projects, Table S2: Codon saturation analysis results, Table S3: ModelFinder’s output, “POWV_Mascot.xml”—the BEAST xml-project (MASCOT package v.1.2.2 required), “POWV.fasta”—the fasta alignment used in the phylogeography analysis.
