# Supplementary material for: Phylogeography and Re-Evaluation of Evolutionary Rate of Powassan Virus Using Complete Genome Data
Source: Biology (Basel). 2021 Dec 6;10(12):1282. doi: 10.3390/biology10121282 (PMC8698833; doi:10.3390/biology10121282)
Supplement: Supplementary file 1 [file biology-10-01282-s001.zip › biology-1448078-supplementary/Table S2.pdf]

1+2 codon positions resuts:

Part I. For a symmetrical tree.

==

Prop. invar. sites	0,7639
Mean H	0,2064
Standard Error	0,0093
Hmax	1,8953
Iss	0,1089
Iss.c	0,8208
T	76,7008
DF	1612
Prob (Two-tailed)	0,0000
95% Lower Limit	0,0907
95% Upper Limit	0,1271

==

Part II. For an extreme asymmetrical (and generally very unlikely) tree.

==

Iss.c	0,6177
T	54,8153
DF	1612
Prob (Two-tailed)	0,0000
95% Lower Limit	0,0907
95% Upper Limit	0,1271

==

Interpretation of results:

	Significant Difference: Yes	Significant Difference: No
Iss < Iss.c	Little saturation	Substantial saturation
Iss > Iss.c	Useless sequences	Very poor for phylogenetics

3rd codon position results:

Part I. For a symmetrical tree.

===

Prop. invar. sites	0,1122
Mean H	0,4875
Standard Error	0,0085
Hmax	1,8936
Iss	0,2575
Iss.c	0,8045
T	64,4748
DF	3029
Prob (Two-tailed)	0,0000
95% Lower Limit	0,2408
95% Upper Limit	0,2741

===

Part II. For an extreme asymmetrical (and generally very unlikely) tree.

===

Iss.c	0,5967
T	39,9893
DF	3029
Prob (Two-tailed)	0,0000
95% Lower Limit	0,2408
95% Upper Limit	0,2741

===

Interpretation of results:

	Significant Difference: Yes	Significant Difference: No
Iss < Iss.c	Little Saturation	Substantial saturation
Iss > Iss.c	Useless sequences	Very poor for phylogenetics
