# Supplementary figures and images for: Phylogeography and Re-Evaluation of Evolutionary Rate of Powassan Virus Using Complete Genome Data
Source: Biology (Basel). 2021 Dec 6;10(12):1282. doi: 10.3390/biology10121282 (PMC8698833; doi:10.3390/biology10121282)

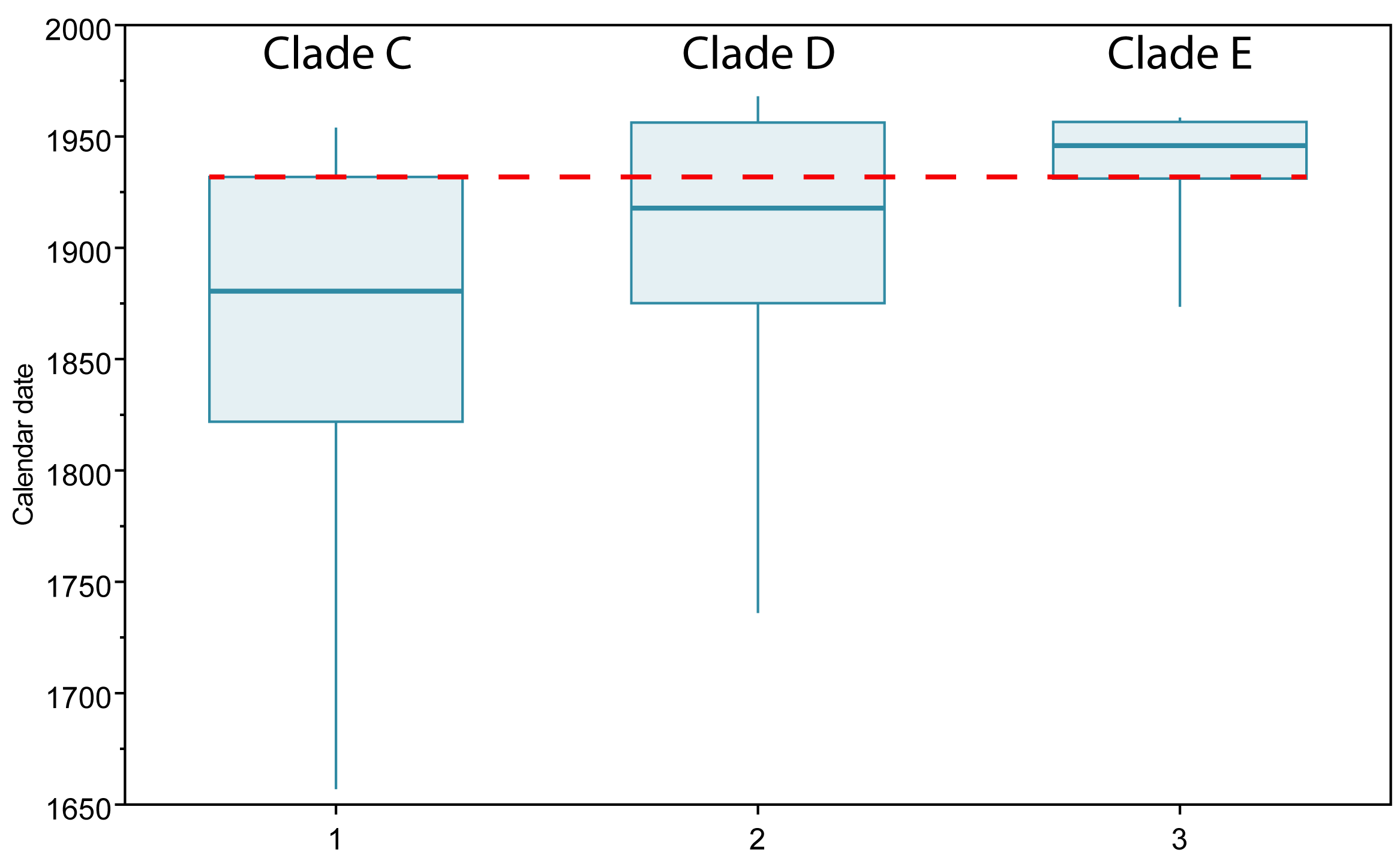

Supplement: Supplementary file 1 [file biology-10-01282-s001.zip › biology-1448078-supplementary/Figure S3.tif]
